# Abstracts of Meeting

**Published:** 1982

**Authors:** 


### Surgical Club of South West England

**Friday, 6th November, 1981**
Gloucester Royal Hospital, Wotton House

**Papers for the South West Surgical Prize for Registrars**

### FLOW MEASUREMENTS IN THE HUMAN COMMON BILE DUCT – A possible new peroperative test of the function of the sphincter of oddi

**N. Setakis, W. Vennart**
**Sponsored by Mr. A. M. Gardner, Torbay Hospital**

The flow rate of normal saline through the human common bile duct into the duodenum has been measured at operation before and after the administration of Glucagon or Probathine in 46 patients (Tables 1 and 2).

A simple experimental model similar to that of Daniel's was used to convert these flow measurements into the diameter of the opening into the duodenum (Figure 1).

An 8F.G. feeding tube inserted into the cystic duct and connected to the barrel of a 20 ml syringe filled with normal saline at body temperature, the syringe was then raised 30 cms above the point where the pressures in the bile duct and reservoir were equal and the time for 10 mls of saline to flow measured. These measurements were repeated after the intravenous injections of 2 mgs Glucagon or 30 mgs Probathine.

Of the 29 patients with Glucagon, five patients had diameter increases of less than 5% – these patients were treated with sphincterotomy or dilatation. Seven patients had unimpacted stones in the duct and as the model predicts the flow was not found to be altered. In one case of acute pancreatitis the diameter of the opening was small, but it increased after the administration of Glucagon. This dilatation of the ampulla with an associated drop in pressure in the bile duct is the proposed mechanism for the immediate relief from pain that patients experience after administration of Glucagon for the treatment of pancreatitis. In a further 17 patients Probathine was found to have little effect on dilating the sphincter and did not inhibit the subsequent dilating effect of Glucagon.

In the absence of impacted stones, the failure of the ampulla to relax is indicative of organic stenosis.

These flow measurements, therefore, together with the administration of Glucagon would seem to have value in the diagnosis of fibrosis of the ampulla.

### THE ACUTE SCROTUM

**David Cranston**
**Sponsored by Mr. Clifford Moisey, Royal United Hospital, Bath**

Testicular torsion is a relatively common surgical emergency often difficult to diagnosis accurately and still leading to a high orchidectomy rate through delay in presentation, diagnosis, referral or operation.

Some testes are of questionable viability at operation and are returned to the scrotum to await the passage of time. Of these some undoubtedly do not survive and atrophy over the succeeding months leading to a higher testicular 'death' rate than the orchiectomy figures would indicate.

Two hundred and fifty-nine patients with an acute scrotum presenting to the Bath Hospitals between 1966-80 are reviewed.

One hundred and fifty had torsion of the testis, 32 (21%) requiring orchidectomy. Thirty-three had torsion of appendix testis, 35 epididymo-orchitis and the remaining 41 (all under 30 years of age) were not explored, the majority being clinically diagnosed as epididymo-orchitis.

Twenty-nine of the 32 requiring orchidectomy experienced a delay of over 12 hours from the onset of symptoms to surgery.

The true testicular atrophy rate is being studied by further review and re-examination. To date 11 out of 22 (50%) of patients with a testis thought to be viable at operation and left in situ, now have an atrophic testis. Six out of 15 (40%) of patients not explored now have an atrophic testis.

We stress that the history and examination are misleading, suggesting epididymo-orchitis when subsequent exploration reveals a torsion. We re-echo the plea that so-called 'epididymo-orchitis' in any patient under 25 is diagnosed on the operating table.

### VESICO-URETERIC REFLUX

Denis G. Calvert, Gloucester

The Paper was based on forty-eight personal surgical cases many of whom had both ureters refluxing. Mortality was nil. Four ureters needed further reimplants due to obstruction.

The current operation being employed is the Cohen type procedure but most of the cases had had the Leadbetter Politano method of reimplantation. The most important criterion as regards surgical intervention was the presence of infection and reflux. The relationship of infection to reflux was discussed. The most serious aspect of the condition is the presence of infection in the kidney of the child and chronic pyelonephritic changes are common and correction of the reflux appears to halt the progress of the pyelonephritic scarring. It is probably safe to watch cases of reflux in adult women who are asymptomatic in the absence of urinary infections, though minor loin pain may be indication for surgical correction in order to relieve symptoms.

Hopes for the future are a less invasive method of investigation than the present micturating cystogram.

### PROSPECTIVE TRIAL OF MAINTENANCE CIMETIDINE AND PROXIMAL GASTRIC VAGOTOMY FOR DUODENAL ULCER

Michael W. L. Gear, Gloucester

In Gloucestershire, long term Cimetidine maintenance treatment has been compared with proximal gastric vagotomy in a randomised trial. However, it has proved difficult to perform the trial because of the enormous usage of Cimetidine by GPs and the low referral rate for surgery.

Forty-two patients referred with long-standing, chronic duodenal ulcer were randomised to treatment, either with maintenance Cimetidine, 400 mg nocte, or proximal gastric vagotomy. All patients were followed up in the clinic at 3-monthly intervals and at check endoscopy 6-monthly or more often if symptoms recurred. Follow up is from one to 5 years, more than half the patients having been followed for over 2 years.

**Results in the Medical Treatment Group:**
Only 46% of the 22 patients treated medically remained healed. Six patients developed recurrent ulcers during treatment and another 7 patients developed recurrent ulcers a short time after stopping treatment.

**Results in the Surgical Treatment Group:**
One patient has developed a recurrent ulcer in this group. All the other patients are graded either Visick I or II.

**Complications:**
Two patients died of unrelated causes, one in the medical and one in the surgical group. One patient on long-term maintenance developed a severe allergic hepatitis, another developed a severe kerato-conjunctivitis which seemed to be worse when the dose of Cimetidine was increased to try and heal the recurrent duodenal ulcer. Three surgical patients developed minor wound complications.

It is concluded that surgery provides a much better long-term protection against recurrent ulcer. Cimetidine is useful in the short term to treat those patients who do not require surgery or in whom surgery would be contra-indicated.

### THE DA SILVA REPAIR FOR MEDIAN AND PARAMEDIAN INCISIONAL HERNIA

John Kilby, Gloucester

The technique for repairing longitudinal incisional hernias by the da Silva method was described and a film of the technique shown. The method is based on the establishment of flaps from the rectus sheaths on both sides creating a non-tension repair which restores the rectus muscles to their original position. The strength of the repair therefore depends on the normal action of the rectus muscles rather than on the materials used. Satisfactory results on 11 cases were reported with a follow up of 6 months to 2 years.

### A NEW OPERATION FOR RECTAL PROLAPSE

H. Thomson, Gloucester

The procedure is based on observing that in prolapse the anterior rectal wall peels off the vagina. This stimulated the idea that a cure might be achieved by pinning it permanently in place.

The patient lies, under GA, in the prone 'jack-knife' position. A Parks speculum gives access. The anterior rectal wall is pushed into view by fingers of the left hand in the vagina, and stitched with thick Dexon to the posterior wall of the vagina, picking up the latter in substantial bites, guided by the vaginal fingers. Interrupted sutures are placed from the apex of the perineal body upwards at centimetre intervals to the highest part of the vagina, and as far laterally as possible

(except at the posterior vaginal fornix where stitching is confined to the mid line to protect the ureters). At the finish the anterior rectal wall looks like a quilt.

The patient goes home the following day with a hydrophilic aperient and advice concerning both the avoidance of straining and the necessity for perineal exercises.

At a recent review 13 of the 23 so treated remained cured (average follow-up 3½ years). Recurrences were associated with large prolapses and demented patients.

The operation may have a place for the moderate prolapse in a sensible woman.

### DOCTORS' BELIEFS AND BIOCHEMICAL REALITY REGARDING RECTAL EXAMINATION AND ACID PHOSPHATASE

**A. S. Daar*, Gloucester**

Although there exists convincing evidence to the contrary, there is widespread belief that it is necessary to delay taking blood for acid phosphatase estimation after rectal examination of the prostate.

We sent a questionnaire to 210 hospital doctors of all grades and specialists at 2 large district hospitals and one University teaching hospital in England asking the following two questions.

1. Is it necessary to delay taking blood for acid phosphatase estimation after rectal examination of the prostate?
2. If 'yes', for how long?

Of the 150 replies received, 69% replied 'Yes' to the question 1 ('positive responders'), 17% did not know, and only 15% believed it was not necessary. Of the positive responders the highest incidence (80%) was amongst the most junior doctors, implying that the belief is still taught in medical schools. Response to question 2 showed that 46% would wait 24-48 hours, and 33% up to one week.

We then carried out a study of the total (TAP) and the prostatic fraction (PF) of the serum acid phosphatase before and at 5 minutes, 1 hour and 24 hours after rectal examination in 36 general surgical patients. There was no change in TAP and similarly, there was no change in PF with rectal examination. We conclude, therefore that it is not necessary to delay taking blood for enzyme activity estimation after rectal examination. The belief that it is necessary however, continues to be taught in medical schools and to young doctors.

*present address:
Nuffield Department of Surgery, University of Oxford

### WOUND SEPSIS AFTER APPENDICECTOMY, Report of a Trial

**D. W. R. Gray, Gloucester**

A prospective randomised trial of prophylactic antibiotics in appendicectomy using delayed primary closure of the wound was undertaken. The antibiotics used were metronidazole and cephradine. Patients were admitted to the trial upon the decision to operate for suspected appendicitis. Half the patients received preoperative prophylactic antibiotics in every case, the other half received antibiotics only on finding severe appendicitis at operation. Severe appendicitis was defined as perforation, gangrene or pus formation, thus if appendicitis was not confirmed, or a mildly inflamed appendix was found, antibiotics would not be given. Patients were followed up until their wounds were healed, and any evidence of infection noted.

In all, 201 patients entered the trial, with follow up completed on 199. No case of wound infection in hospital occurred in either group. Prolonged drainage from the wound as an out patient occurred in approximately 15% of cases in both groups. This drainage required little more than regular District Nurse dressing it. It is possible that this drainage is an effect of delayed primary closure technique. Antibiotics given prophylactically appear to have no advantage when delayed primary closure technique is used.

### AUTOSUTURES IN GLOUCESTER

**I. M. R. Lowden, Gloucester**

The results of a retrospective survey of operations carried out using Autosutures were presented. The study covered a period of six years, and included all upper Gastro-intestinal operations carried out in this period. The complications following the use of Autosuture as compared with those after standard techniques were studied. There was no significant difference between the two groups. The technique of forming a gastro-enterostomy was demonstrated using slides, and various technical points in the use of Autosutures in upper Gastro-intestinal Surgery were discussed.

### THE EFFICIENCY OF STILBOESTROL IN PREVENTING ERECTION

**S. P. J. Kay, Gloucester**

It is accepted practice in many centres to prescribe stilboestrol peri-operatively for adult patients undergoing penile surgery in the belief that disruptive painful erections will be prevented. In view of the drugs dangers however, its value therein seemed worth investigating. Its ability to suppress erections, either spontaneous or stimulated, was examined in five healthy young male surgeons.

The trial was double-blind, and compared the effect of stilboestrol in two standard doses (1 mg t.d.s and 5 mg t.d.s.) with a placebo, and so involved each participant in four separate four day courses, two with the active preparation, and two with placebo. Although blind, the trial was organised so that placebo and stilboestrol courses alternated, with a nine day gap between each.

Results were reported by anonymous questionnaire. Erection patterns were measured during the treatment days and the subsequent two days. An attempt at erection was required daily.

No difference in total number of erections, spontaneous or stimulated, was found between stilboestrol in either dose or placebo. Neither did the erection pattern alter from day to day within the trial period.

The value of stilboestrol over short periods in preventing erection remains unproven. Thus we suggest its routine use for this purpose be discontinued.

### A WIND INSTRUMENT

**Hamish Thomson, Gloucester**

To conclude the session our local secretary who had previously impressed us with his proctological expertise in describing his new operation for rectal prolapse further enhanced his reputation by showing how the sigmoidoscope could be played as a musical instrument. With the aid of a brass mouthpiece he gave a fairly accurate rendering of the 'Internationale'.

M.G.W. (Ed.)

### Saturday, 7th November, 1981
Postgraduate Medical Centre, Cheltenham

### CRYOSURGERY FOR THE URETHRAL SYNDROME

**Peter Boreham, Cheltenham**

Women with frequency and dysuria, either constantly or in attacks, in whom stones, tumours, tuberculosis and other organic diseases have been excluded, form a great burden in any urological department. They have been treated with mixed success by various means in the past. A technique has been developed for treating them by cryo-surgery using a special guarded applicator on a Keymed cyroprobe. It can be done under local or general anaesthesia. An initial control series of 35 cases was presented, and the results in the last 150. The improvement in symptoms is at least as good as with other treatment and it causes much less discomfort, bleeding or other complications.

### EXPLORATION OF THE COMMON BILE DUCT – MORTALITY AND MORBIDITY

**Christo G. Zouves**

Cholecystectomy is the commonest elective abdominal operation performed in this country, and between 10 and 49 per cent of these patients undergo exploration of their common bile ducts (Le Quesne 1964). In a retrospective study of 390 cholecystectomies performed over a two year period 116 or 23 per cent underwent exploration of common bile duct.

Exploration was by the supra-duodenal approach using a T-tube in 60 (52%), the trans-duodenal approach in 43 (37%) and by a combined approach in 10 (8%). Of the patients explored 76% had demonstrable stones and approximately half of the explorations were done by Consultants, the rest by Registrars and Consultants did more negative explorations.

The mortality in the group undergoing only cholecystectomy was 1.8%, while in those in whom exploration was carried out supra-duodenally it was 3.3%, trans-duodenally 14.0% and zero in those undergoing the combined procedure.

The causes of death in the cholecystectomy only group could not be related to the surgical procedure per se, as was the case in the supra-duodenal group (pulmonary embolus, myocardial infarction, aspiration pneumonia and CVA), while in the group of 5 patients who died after trans-duodenal exploration 3 were known to have suffered some accident at the site of anastomosis which probably contributed to their demise.

Morbidity in the form of pancreatitis, bile leaks, renal failure and chest infections were also more common in the group explored trans-duodenally.

There was no significant difference in the duration of Hospital stay which was about 14 days in the explored groups. Three patients were known to have retained stones after supra-duodenal exploration and one patient underwent successful Dormia basket extraction, the second passed the stone after flushing with heparin and saline solution and the third patient had a failed Dormia extraction and was observed and is well after two years.

### CAROTID SURGERY IN A DISTRICT GENERAL HOSPITAL

**John Fairgrieve, Cheltenham**

The carotid surgical experience of Cheltenham General Hospital over a 13 year period (1968-81) is presented. This includes 42 operations for stenosis, and 12 further operations for carotid body tumour, carotid aneurysm, subclavian steal syndrome and trauma to the internal carotid artery. The operative techniques and complications are briefly discussed and reasons advanced for a more agressive approach to the problems of extra-cerebral carotid disease in the country.

*(Full text in preceding number – Vol.96 iii/iv)*

### IN-SITU FEMORO-POPLITEAL (AND DISTAL) VEIN BYPASS GRAFTS FOR LIMB SALVAGE – EXPERIENCE OF 50 CASES IN A DISTRICT GENERAL HOSPITAL

**M. Denton, J. Fairgrieve, Cheltenham**

This paper reviews our experience of the first 50 cases of 'in-situ' technique of femoro-popliteal or distal bypass using autogenous saphenous vein. The procedures were performed for limb salvage over a period of 3 years with an overall limb salvage rate of 72% and an 18 month patency and salvage rate of 78%. Only 1 patient died within 30 days of operation, but a further 4 died of causes unrelated to their surgery.

We have reviewed the graft patency in relation to indication for surgery, angiographic evidence of extent of disease, size of graft, site of distal anastomosis and complications of surgery. While all these factors influence surgery, the severity of distal disease is the most important factor in determining outcome.

The in-situ method is no more time consuming than the reversed vein method of bypass, and the intraluminal valve disruptors make valvotomy safe, quick and reliable. But the in-situ method has definite benefits by allowing a small vein (2.5-4 mm) to be used and in facilitating anastomosis to a calf vessel.

### SOLITARY THYROID NODULES

**Stephen Haynes, Cheltenham**

120 operations on the thyroid were reviewed. Two groups were identified with high rates of malignancy. 20% of cases clinically diagnosed as non-toxic modular goitre were subsequently shown to be malignant. 20% of solitary thyroid nodules were also malignant. Several cases were found to have separate nodules of apparently normal thyroid tissue adjacent to the main gland, these being larger than 1 cm in diameter and situated lateral to and below the gland. Frozen section confirmed thyroid tissue and the assumption was made of metastatic nodes; subsequent histology of the total thyroid failed to confirm malignancy. It is therefore suggested that 'lateral aberrant thyroid' reported of old does exist and should be recognised as not always indicating malignancy. Non-toxic nodular goitre should carry the suspicion of malignancy, as should solitary thyroid swellings, the latter justifying the term Thyroid Solitoma.

### ISOTOPIC SCANNING OF THE SCROTUM

**W. E. G. Thomas, Bristol**

Urgent testicular scans using a bolus intravenous injection of 5mCi $^{99}Tc^{m}$ pertechnetate have been studied in 80 patients presenting with acute testicular pain. When the dynamic gradient of uptake of isotope and static pattern were identical on each side of the scrotum, the scans were interpreted as normal. In these cases the mean gradient ratio was 0.99 (S.D. $\pm$ 0.15) but rose to 1.5 (S.D. $\pm$ 0.31) in acute inflammation, and fell to 0.56 (S.D. $\pm$ 0.05) in acute torsion.

Of 24 normal scans, 19 cases were not explored and in 13 no clinical abnormality was found. One case had suffered from trauma, 2 had epididymal cysts and 3 had varicoceles. Of the 5 who came to surgery, 2 testes were normal, and 3 had torsion of the appendix testis.

Increased uptake occurred in 37 patients, 30 of whom had clinical evidence of epididymo-orchitis. Of the remaining 7, 4 had testicular tumours, 1 had suffered trauma, and 2 had a resolved torsion, the increased uptake representing reactive hyperaemia.

Seven patients presented with diminished uptake and the diagnosis of acute testicular torsion was confirmed by immediate operation.

Twelve patients presented with a 'halo sign' on the static image. Of these, 9 had chronic testicular torsion with a history of over 48 hours, and 3 had tumours.

Radionuclide scanning is therefore a rapid and effective method of evaluating testicular blood flow.

### THE ROLE OF PERITONEAL LAVAGE IN THE PREDICTION AND TREATMENT OF SEVERE ACUTE PANCREATITIS

**M. J. Cooper, R. C. N. Williamson, Bristol and A. V. Pollack, Scarborough**

Peritoneal lavage has been reported to improve survival in patients with severe pancreatitis and to enable an accurate prediction of severity to be made. To investigate these reports 87 consecutive patients with acute pancreatitis (serum amylase > 2000 iu/L Phadebas) were entered into a prospective controlled trial to determine the efficiency of four predictive criteria of severity. There were 44 males and 43 females with a mean age of 61.4 years, the majority (64%) having gallstone related pancreatitis.

The predictive criteria on admission were shock (BP > 100 mm Hg systolic), hypoxaemia ($PaO_2$ > 7.98 kPa), hypocalcaemia (> 2.00 mMol/L) and toxic peritoneal broth. Of 23 patients who fulfilled one or more criteria, ten died (43%) and five developed other major complications (22%). Two of 64 with predicted mild disease died (3%) and four developed major complications (6%). The overall accuracy of these predictive criteria is 84%. Twenty-three patients with predicted severe disease were randomised to receive therapeutic lavage with hourly cycles of 2 L dialysate (9 patients) or to act as controls (14 patients). The results are shown in the table.

| | *Control* | *Lavage* |
|---|---|---|
| Benign course | 4 | 3 |
| Major complications | 3 | 3 |
| Death | 7 | 3 |

The four predictive criteria provide an accurate guide to the likely severity of an attack of acute pancreatitis, but the value of peritoneal lavage as a therapeutic modality remains unproven.

